# New mothers’ struggles to love their child. An interpretative synthesis of qualitative studies

**DOI:** 10.1080/17482631.2018.1490621

**Published:** 2018-07-05

**Authors:** Idun Røseth, Rob Bongaardt, Anne Lyberg, Eva Sommerseth, Bente Dahl

**Affiliations:** a Department of Child and Adolescent Psychiatry, Telemark Hospital, Skien, Norway; b Department of Health, Social, and Welfare Studies, Faculty of Health and Social Sciences, University of South-Eastern Norway, Porsgrunn, Norway; c The Centre for Women’s, Family and Child Health, Department of Nursing and Health, Faculty of Health and Social Sciences, University of South-Eastern Norway, Vestfold, Norway

**Keywords:** Disturbed maternal affection, motherly love, interpretive synthesis, qualitative studies, mother–child interaction

## Abstract

**Purpose**: New mothers may question the nature of their motherly love after the birth. Most mothers find that feelings of affection come within a week from birth. However, some mothers are still struggling with this after many months. Many studies place strong emphasis on the importance of maternal affection for the development of the child. Few studies look into mothers’ experiences when maternal affection or love remains a struggle. **Method:**We present an interpretative synthesis based on a systematic analysis of five qualitative studies that report findings related to mothers’ stated inability to exhibit maternal affection. **Result:**In answer to our question “what characterizes the experiences of women who struggle with, or are unable to exhibit, maternal affection after birth”, we identified the uncertainty involved in imagining the unborn child, birth and maternal future, birth as a disillusionment, and the ensuing process of decreasing agency and increasing alienation. Especially a traumatic birth may lead to disillusionment. **Conclusion:** Health care workers and research can support a mother’s positive resolution of her struggle by promoting realistic and more open expectations for maternal affection as well as her sense of agency and ownership during birth and the early mother–child relationship.

It is not uncommon that new mothers question the nature of their motherly love after birth. One study revealed that as many as 40% of first-time and 25% of second-time mothers recalled feeling indifference when holding their baby for the first time (Robson & Kumar, 1980). Most mothers find that feelings of affection come within a week from birth. However, some mothers are still struggling with this after many months (Righetti-Veltema, Conne-Perréard, Bousquet, & Manzano, 2002). In the general population of postnatal women, two prospective studies reported rates of poor mother–child bonding to be 7.1% at two weeks (Reck et al., 2006) and 8.9% at 12 weeks (Taylor, Atkins, Kumar, Adams, & Glover, 2005). A small percentage may even have hostile feelings towards their infant (Brocketing, Aucamp, & Fraser, 2006). Mothers who experience this lack of affection often feel like bad mothers and have strong feelings of guilt and shame (Røseth, Binder, & Malt, 2011). The mother’s emotional state may affect the child even around the time of birth (Della Vedova, Duccschi, Cesana, & Imbasciati, 2011; Diego, Field, & Hermandez-Reif, 2005). An early nurturing and affectionate maternal bond builds resilience against psychological challenges that children may meet (Miller, Kramer, Warner, Wickramaratne, & Weissman, 1997). Lack of maternal warmth, especially when connected to controlling caregiving, has been found to be related to an increase in psychological and social problems in children later in life (e.g., Dalsant, Truzzi, Setoh, & Esposito, 2015; Della Vedova et al., 2011).

**Figure 1.**
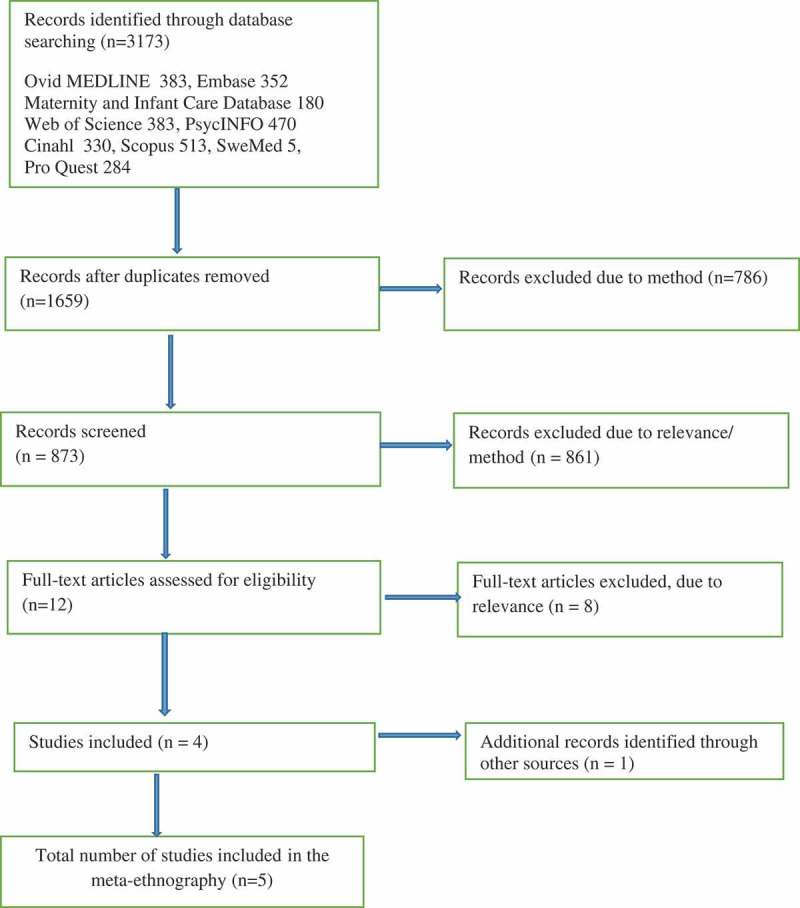
Data search using the PRISMA flow diagram (Moher, Liberati, Tetzlaff, & Altman, 2009).

The results of these studies all strongly emphasize the importance of maternal affection for the development of the child. These studies are based on screening questionnaires (Brockington, Fraser, & Wilson, 2006; Taylor et al., 2005) and retrospective data on maternal caregiving. They typically reveal a correlation between the participants’ memories of their mothers’ maternal affection and symptoms of psychological and social problems, but do not present any further insights or explanation of these findings as experienced by mothers.

The purpose of this article is to contribute to the research field with in-depth knowledge about mothers’ experience of having problems with maternal affection or love. To this end, we present an interpretative synthesis based on a systematic review of qualitative studies that report findings related to mothers’ stated inability to exhibit maternal affection. The research question of our study is “what characterizes the experiences of women who struggle with, or are unable to exhibit, maternal affection after birth?”

## Method

The method of our study is best described as an interpretative synthesis of qualitative research (Weed, 2005). First, to systematize the collection and analysis of the data, we took inspiration from Noblit and Hare’s (1988) meta-ethnography to synthesize qualitative research studies. Meta-ethnography includes a systematic search of articles that report on qualitative studies about one’s topic of interest and is suited to the synthesis of a small number of studies. It scrutinizes the articles’ various sections for insights into the research topic and the context in which these insights arose (Bondas & Hall, 2007; Nicoll, 2016). Then, in creating the final synthesis, we were guided by Van Manen’s approach to hermeneutic phenomenological writing (Saevi, 2013; Van Manen, 1997), which stresses an ongoing exploration of the words and phrasings that capture the felt sense of the lived experience in the best way. Finally, to deepen our understanding of this experience as described in the synthesis, we followed up with a dialogue between our findings and phenomenological existential and attachment-based research. We preferred to call this a “dialogue” rather than a “discussion”, because the latter connotes a division into parts, while we wished to stress the dialogic relationship between the parties involved. In the following, we describe the steps we have taken to synthesize qualitative research articles that to some degree described experiences of women who are unable or struggle to exhibit maternal affection after birth.

### Search strategy and inclusion criteria

After formulating the research question, we specified, in collaboration with an expert librarian, search terms and online databases to be used. The key search terms “maternal affection”, “bonding”, “love”, “attachment”, and “identity” were used with the limitations “qualitative”, “year 2007-present”, and “peer reviewed”. The databases searched were Ovid MEDLINE, Embase, PsycINFO, Maternity and Infant Care Database, Cinahl, Web of Science, ProQuest, Scopus and SveMed+. Qualitative peer-reviewed papers published in English or Scandinavian languages were included. These concerned the experiences of first-time and multiparous mothers, with both normal and complicated labour. Excluded were quantitative studies, mixed methods studies, literature reviews, methodological papers, conceptual/theoretical papers, conference abstracts and dissertations. In addition, we excluded papers that examined phenomena involving mental disorders in mothers, the experience of mothers under 18 years of age and mothers of sick or handicapped infants. Studies concerning mothers from developing countries were excluded, except for studies conducted in Chile, Brazil or South Africa.

### Search outcome

The initial search identified 3173 articles, 1659 after removing duplicates. A team of five researchers screened the articles as follows: from the Endnote file of the returned records, we excluded articles that did not use the term qualitative in the title, key words, or abstract. Then we reviewed the title, abstract and key words of the remaining 873 papers and excluded papers that were methodologically or thematically irrelevant. After this review, 12 papers were obtained in full text and scrutinized according to the inclusion and exclusion criteria. Eight papers were found irrelevant; while these articles concerning related themes such a mother’s identity and depression after childbirth, they did not include any descriptions or discussion of experiences of women who struggle with, or are unable to exhibit, maternal affection after birth. The remaining four articles were considered eligible and included in the study. One additional relevant article was identified through a manual search of reference lists. (See Figure 1). An additional manual search of three journals (BMC Pregnancy and Childbirth, Journal of Prenatal & Perinatal Psychology and Health and International Journal of Qualitative Studies on Health and Well-Being) did not yield any relevant articles in addition to the five already found.

### Critical assessment of the included articles

The five articles were assessed independently by two authors (ES and AL). They applied “The Joanna Briggs Institute Critical Appraisal Checklist for Qualitative Research” (Lockwood, Munn, & Porritt, 2015) to each article and subsequently discussed their assessment to obtain agreement. The checklist contains 10 criteria of which seven address the congruity between philosophical perspective, research methodology, methods used, data representation and analysis, participant voice, interpretation of data, and conclusion. All the studies showed this congruity. Two criteria concern authors’ statements on their cultural or theoretical background and how this may have influenced research. Such statements were not present in all articles. One article did not inform about formally acquired research ethical approval, i.e., the tenth quality criterion. Based on these assessments, the eligible studies were considered to be of medium or good quality and were all used in our study. The characteristics of the studies are described in more detail below.

### Characteristics of the included studies

The studies reported on in the articles were conducted in the U.S.A., U.K., Australia, Sweden and South Africa between 2007 and 2014 (Table I). They included 63 women who were unable to or struggled to exhibit maternal affection. Sample size varied between 5 and 21. Data were collected by face-to-face and telephone interviews. The studies used various methodological approaches, such as phenomenology and thematic analysis. All studies analysed descriptions of women’s lived experiences of bonding pre- and postnatally and how the women made sense of emotional distress in the first year after birth. Studies also described the impact of complications during labour on the women’s early mothering experiences. The studies provided a variety of examples describing women’s experiences of problems with exhibiting maternal affection. Table I presents an overview.

**Table I.** Characteristic of the included qualitative studies.

Author Year Country	Aims & research questions	Ethics	Participants characteristics	Sample method & recruitment	Data collection & data analysis
Barrack, 2007 USA	To understand parents’ experiences of bonding with their babies in utero and after birthTo discover the relevance of a prenatal and perinatal psychology (PPN) based bonding class in this process	No information	Five couples, four married, one unmarried	Sample recruited from prenatal parenting classes, method not described	Semi-structured interviews, using video and audiotape conducted 2–6 months after birthPhenomenological and portraiture analysis
Coates et al., 2014 UK	To explore how women experienced and made sense of the range of emotional distress states in the first postnatal year	Ethical approval from the university Research Governance Committee and NCT Research Office	17 women aged 23–42 years who experienced psychological problems in the first year after birth	Opportunistic. Advertisements placed on relevant websites, in local National Childbirth Trust newsletters, and through instructors at relevant antenatal and postnatal classes	Semi-structured face-to-face or telephone interviewsInterpretative phenomenological analysis (IPA)
Elmir et al., 2011Australia	To describe the experiences of women who had an emergency hysterectomy following a severe postpartum hemorrhage and the impact on early mothering experiences	Approval from the human research ethics committee at the university	21 Australian women aged 26–57 years who had a postpartum hemorrhage/emergency hysterectomy	Recruitment proceeded through media release, flyers and posters being disseminated around university campuses and public places	Semi-structured, face-to-face, telephone and email internet interviews Software NVIVO using thematic analysis
Nystedt et al., 2008 Sweden	To explore women’s experience of becoming a mother after prolonged labour	Approval by the Ethics Committee of University Medical Faculty	Ten women with prolonged labour (first child)	Participants were identified through medical records, and recruited by invitation letter	Individual interviews Thematic content analysis
van Reenen & van Rensburg, 2013 South-Africa.	To explore the subjective experiences and perceptions of women who delivered their babies by an unplanned Cesarean section and their influence on maternal attachment representations and mothers’ relationship with their babies	Approval by relevant Ethics Committee	10 women who had delivered babies by unplanned Caesarian section	Snowball sampling	In-depth phenomenological interviewsThematic content analysis.

### Analysis and synthesis

The process of analysis involved three major phases: identifying the meanings of women’s experiences as reported in each of the five studies, bringing together shared meanings between the five studies as facets of struggling or being unable to exhibit maternal affection after birth, and synthesizing these facets in a coherent account.

Here follows a more detailed account of how the analysis unfolded. We conducted a critical, meticulous and iterative analysis of the five articles separately. We read and reread each article, carefully noting the meaning in the concepts, themes, quotes and phrases that pertained to our research question. We also searched for meanings that were not directly described or acknowledged by the author and used the whole article as an important context for our analysis. This critical analysis revealed implicit meanings, which helped clarify the connotations of concepts in the article and sometimes resulted in nuancing the meanings of an account as interpreted by the authors (Nicoll, 2016). We took note of important meanings and formulated initial ideas about the relationship between them, both within and between the articles. In this process, we included generous amounts of text, including participants’ quotations as well as the original authors’ interpretations. The initial ideas about relationships between the articles were then transformed into facets of the experience at hand. As these facets displayed a coherent chronological order, we used this order as the basis for writing the synthesis.

### Limitations

When determining a study’s transferability, the range of empirical variation in the sample must be taken into consideration (Malterud, 2001). In this study, the sample included 63 women from five different countries, the majority being Caucasian but representing women of different status and age. We are aware that the inclusion of women from non-Western cultures may have provided a greater variety of data, thereby strengthening transferability. However, this was beyond the scope of our study. Three of the included articles address complicated labour situations as the context of the mother’s struggle with maternal affection. We included these articles because descriptions of the phenomenon of our interest are present in these articles, even though they were strongly intertwined with the context in which they emerged. In service of our analysis we thereby inverted the respective roles of context and thematic focus in these articles to highlight maternal affection. In the results we explicate how context and thematic focus stand in relation to each other. We acknowledge that if the struggle with maternal affection is studied in other contexts, more perspectives can come to the fore. This would allow for nuancing further the structure of the phenomenon as we describe it below.

## Results

We identified several facets of the struggle or inability to exhibit maternal affection after birth. These are the uncertainty involved in imagining the unborn child, birth and maternal future, birth as a disillusionment, and the ensuing process of decreasing agency and increasing alienation. We also describe the positive resolution that some mothers experienced after this troublesome period. Below we include quotes in italics citing the mothers’ verbalization of their experiences in the original studies.

### Imagining the unborn child, birth and maternal future

While the child is in the womb, the mother can imagine what her child is like, how giving birth will be, and how she will feel and act as a mother. As the mother feels a connection to her child, images of the future may turn into concrete expectations about the new, emerging life. These expectations are embedded in excitement along with fear and nervousness, which set the emotional stage for what is to come after giving birth. What counts as meaningful or uncertain is fed by earlier experiences. The maternal future is informed by past experiences. “Mothers and fathers experience this journey in different ways, as the lens through which each individual views life is tinted by that person’s travel so far” (Barrack, 2007, p. 63). Also others, such as health care professionals, set the stage through their explanations of what is to come after birth or through their failure to provide any explanation.

Three studies reported specifically on prenatal experiences of the mother’s relation to the foetus (Barrack, 2007; Nystedt, Högberg, & Lundman, 2008; van Reenen & van Rensburg, 2013). Mothers in Barrack’s (2007) and van Reenen and van Rensburg’s (2014) studies reported developing a positive bond to the imagined child. Maternal affection was enhanced by several events, of which the most important was the growing awareness of the foetus as a separate person with its own subjectivity.

*[The bond with the baby twins] was really strong and once I could actually feel them move, I could really start bonding with them more. I think from the moment I got pregnant, there was a certain amount of bonding going on because I definitely felt like I wasn’t alone anymore. I knew there were these two little souls in me, and so I felt that was there from the beginning, but it grew as I grew, definitely* (Barrack, 2007, p. 63).

Pregnancy was described by some mothers as a spiritual, special bonding process. As one mother said, pregnancy was *“personal, intimate, just between [me and my baby]”* (van Reenen & van Rensburg, 2013, p. 270). They endowed the imagined child with rather complex intentions, thoughts and feelings, or even telepathic abilities.

*[S]he would kick and be like I’m okay, I’m a strong girl … So it was really kind of neat, anytime I would have a thought of worry, she would like back it up. Just with either thought through my own brain, or she would kick or she would move or she would just let me know, hey, I’m cool. Don’t worry. Everything’s good* (Barrack, 2007, p. 64).

The physical connection with the child as it materializes in the changing body as well as the movements of the foetus facilitated the development of maternal affection as the mothers became more aware of the child as a separate existence.

Some mothers reported making detailed plans about birth and maternal life (Barrack, 2007; van Reenen & van Rensburg, 2013). They often had an outspoken desire to be able to birth naturally without complications. Some imagined giving birth naturally as significant for “bonding” (van Reenen & van Rensburg, 2013, p. 270). A basic assumption was that “*[i]t’s what my body was designed to do*”, yet they also actively worked with their body to enhance its capacity for natural birth. This desire underscored that they regarded birth as an accomplishment, comparable to a final examination.

In Nystedt et al.’s (2008) study, some of the mothers experienced a far more detached relationship with the unborn child. They remembered being scared about having a child, doubting whether they could fulfil the maternal role, and whether they actually wanted the child.

*I was terrified [of being not able to care for and look after a child] and I would like to think that I’m not going to manage this, and I’ve always been a bit suspicious of children, as well* (p. 255).

Similarly, Barrack (2007) reported in her findings that feeling unprepared for pregnancy, not knowing the sex of the child and the experience of anxiety limited mothers’ ability to bond.

### Birth as a disillusionment

With childbirth, the mother enters a new life situation. For the mothers who had detailed plans and expectations about the birth process, the actual birth experience clashed with her expectations of giving birth, especially when there were complications (Barrack, 2007; Coates, Ayers, & de Visser, 2014; Elmir, Scmied, Wilkes, & Jackson, 2011; Nystedt et al., 2008; van Reenen & van Rensburg, 2013). When “complications arose and the birth did not proceed as intended, parents often felt like failures” (Barrack, 2007, p. 64). *“I was supposed to do it [giving birth]; me, not the doctors”* (van Reenen & van Rensburg, 2013, p. 270). The medical procedure and sedation were experienced by women as a loss of agency as they became passive spectators. “*You are just sitting there and your baby is being born for you”* (van Reenen & van Rensburg, 2013, p. 270).

Women who had specific expectations about a natural birth, struggled when complications arose, and they often felt like failures. “For many parents who spent months planning a trip to the tropics, they feel like they have inadvertently been dropped off in the arctic” (Barrack, 2007, p. 65). The clash between expectations and reality then easily led to negative feelings of inadequacy, loss and guilt. Some of the women who experienced an emergency hysterectomy perceived this as a form of failure because of all the trauma and distress they believed they had inflicted on their baby.

*I had this guilt, and probably still do a little bit that my body let me and her down because she came so early, and you kind of have this guilt that you know, you somehow have caused your baby to suffer* (Coates et al., 2014, p. 6).

When her uterus “gave up”, she would blame her body for opposing her will. Does this set the stage for the time ahead, she wondered. The mother grieved the loss of something perfect, namely a good birth experience, and wished for clarity about what had happened during birth to come to terms with her expectations and reset these if possible.

These negative birth experiences were often attributed to properties of the self (Barrack, 2007; Coates et al., 2014; Elmir et al., 2011). Mothers were consumed by negative feelings about self, and experienced guilt and sadness. These negative feelings of self influenced and delayed the development of maternal affection.

*I felt very guilty for not being there in the initial phase, post birth, to breastfeed him and bond with him. I initially felt a sort of distance between us because of this*(Elmir et al., 2011,*p. 1123).*

Many of the women who experienced giving birth as traumatic described the need for a resolution of the birth experience in order to continue with their life as mothers.

[Labour and birth] was just nothing like what I’d imagined so I just felt … like just at a disadvantage. Like I’d been thwarted all the way through and … something was taken away from me so I felt like couldn’t really recover, to get back to square one, how I wanted to start out with this new life (Coates et al., 2014, p. 5–6).

Coming to terms with a negative birth experience involved exploring what happened to them during birth, using time and energy to process the experience.

I don’t know what happened when he went into the Special Care. I never managed to find out, so I’m quite keen to find out exactly what happened, and I’m hoping that will just put a lid on it to be honest, and put it to bed (Coates, Ayers & de Visser, 2014, P. 6).

### Decreased agency and increased alienation

A troublesome start due to birth complications and medical procedures often led to loss of time to get to know the pace and needs of the child. This clashed with the women’s belief that the initial postpartum time was an important period to develop a relationship with the baby, and that the mother should be the first to hold the baby (Elmir et al., 2011). The physical complications of a difficult birth affected the mother’s ability to bond with her baby, especially when negative self-attributions arose. The women described a feeling of detachment or alienation; they felt somewhat removed from the baby, motherhood, or their whole life (Barrack, 2007; Coates et al., 2014; Elmir et al., 2011; Nystedt et al., 2008; van Reenen & van Rensburg, 2013). This sense of detachment was stronger than just feeling unhappy or worried.

*As soon as she was born I didn’t feel right, like I didn’t have a connection with her … I felt like it was someone else’s baby I was holding, it was really weird* (Coates et al., 2014, p. 5).

One mother who experienced a prolonged inability to feel maternal affection described strong frustration and negative feelings towards the baby:

*I had lost that bond with her [the baby] until she was one… I came so close to strangling the kid while she was asleep, it got to a point where my husband would say stay in bed, I’ll go get her because he just knew I was cracking* (Elmir et al., 2011, p. 1124).

When the mothers did not experience the anticipated maternal affections, they often felt guilt and shame, hiding their feelings.

*I felt so guilty and ashamed. And I don’t even think that I talked to [the father] about it, because aren’t you just automatically supposed to feel a bond with your babies? I almost felt more bonded with them when they were inside me, in a way* (Barrack, 2007, p. 67).

The experience of loss of control or agency during birth led to a feeling of detachment or alienation (Barrack, 2007; Coates et al., 2014; Elmir et al., 2011; Nystedt et al., 2008; van Reenen & van Rensburg, 2013).) Some mothers who were ill for a long time after birth, which hindered active participation in the everyday care of the baby, struggled with a feeling of disconnection. “*Walking her, carrying her, moving, sitting, coughing, sneezing, laughing, was all stuff that I couldn’t do… I felt useless”* (van Reenen & van Rensburg, 2013, p. 271). Not being able to care for the infant left the women with a feeling of emptiness and alienation; *[m]y husband bonded with my daughter more than I did. Because I couldn’t get up. I couldn’t get around”* (Elmir et al., 2011, p. 1124).

### Growth of motherly love

As they spent time with the baby, some mothers felt more at home in the situation and caring for the baby became easier. These mothers described that embodied contact with the baby was important when developing maternal affection; holding, cuddling, talking, carrying, bathing, feeding, and sleeping with their infants promoted feelings of being “bonded” (Barrack, 2007; Coates et al., 2014). They gradually developed a relationship and an emotional connection with the child, “a rhythmic dance that slowly tightened the connecting chord between parent and child” (Barrack, 2007, p. 68). As the baby started to communicate more actively, “to make sounds, crawl to them, hug and kiss them, the bond was cemented into their being” (Barrack, 2007, p.68). This was a process where the maternal affections grew with time, enhanced by the baby’s increased ability to communicate with the mother and its emergence as a distinct personality.

Women described maternal affection as an intimate, emotional and embodied connectedness to the child. In Coates et al.’s (2014) study, the mothers mentioned breast-feeding as the single most influential experience in promoting a maternal emotional connection. Often the women felt guilty for all the trauma and distress they believed they had inflicted on the baby and used terms such as “driven”, “determined” or “desperate” to succeed in breastfeeding (Coates et al., 2014; Elmir et al., 2011).

*I breastfed at day two or three… I was so driven to feed my baby, because of the whole stuff up, I want to succeed at this because I failed him*(Elmir et al., 2011,*p. 1123)*.

Women who had experienced a troubled birth perceived breastfeeding as a form of compensation for the negative experience.

### Synthesis of experienced meanings of struggling or being unable to exhibit maternal affection after birth

While pregnancy is certain, giving birth, motherhood and the child are clouded in uncertainty. The void of uncertainty is filled by imagining how giving birth, motherhood and the child will be. The woman’s imagination is fed by earlier personal experiences, information from her partner, family, friends and health care professionals, and especially by the mother’s awareness and sensations of the child in her womb. Between the mothers in the studies, the futures they imagine range from perfect to natural to terrifying. This range denotes the ambiguity that pregnancy implies, understood as uncertainty filled with imagination of possible future scenarios. Many mothers expect birth and the first encounters with the child to provide the resolution of this ambiguity. However, this resolution may not come as expected, and one of two other unanticipated scenarios may play out. In the first scenario, ambiguity transforms into ambivalence; while birth has passed, the child is born and motherhood has materialized, maternal, loving feelings do not take shape. Shapeless, this ambivalence is about not knowing where to turn for any certainty about how to live life as a mother with an unknown child and a de-owned body. Here, the relationship to the child is one of remoteness. Shame may be the accompanying feeling. In the second scenario, ambiguity is resolved but negatively, making reality clash with any anticipated scenario. When the mother’s body did not perform naturally or perfectly as anticipated, trauma and distress occupy the space where the emotional connection should have formed. The mother seems more occupied with what happened in the recent past and how to repair that in the future than with the here-and-now of being with her child. Guilt may be the accompanying feeling. All in all, especially a traumatic birth may lead to disillusionment when accompanied by a reduced capacity to adapt to difficult, unexpected situations. Yet, as the child’s ability to communicate grows and the mother regains her agency through breastfeeding and other daily caregiving activities, the rhythm of daily life between child and mother may synchronize, gradually strengthening maternal affection.

## Dialogue

How does maternal love “take hold” as a feeling in the mother? And why do some mothers struggle to develop these feelings? In our synthesis, the first evidence of maternal affection is described in pregnancy, when many mothers experience an increasing emotional connection with the imagined child. Mothers tell about the development of an intimate relationship with the foetus, which they endow with quite complex intentions, thoughts, and feelings. The mother has fantasies about giving birth, how the child is developing, and how she and her partner will act as parents. These fantasies play a major role in preparations for the birth and postpartum period (Raphaell-Leff, 1991).

The connection between mothers and their foetus increases as it starts to move inside the womb, heightening the mother’s awareness of her child as a separate subject. Similarly, after birth, the mothers often describe an increase in maternal affection parallel to the infant’s growing ability to act and participate as a subject in a common world. Thus, the mother’s affection grows with the increasing separation of herself and her child as individual subjects. Buber (1999) described this paradox by pointing out that for two individuals to have a connection, they must put each other at a distance. Relationship requires distance, as Buber underscores. However, distance does not necessarily lead to a relationship. As humans, we choose the extent to which we bridge this distance, where the other is “becoming a self with me” (Buber, 1999, p. 15). This process of making the other present as a self is dependent on our capacity to envision what the other perceives, feels, wishes or thinks, and also on the other experiencing that we show this capacity. Thus, insofar as the mother accepts and confirms the child as a separate subject, the child can develop and “become a self”. Reciprocally, the increasing ability of the child to reciprocate and confirm the mother’s intentional acts will allow the latter to experience “becoming a self (a mother)” along with her child.

The common cultural conception of maternal love is that of a gendered biological force called maternal instinct, or bonding (Billings, 1995; Klaus & Kennell, 1976; Madrid, Skolek, & Shapiro, 2016). For many mothers, this rings true as their maternal feelings are strongly present from the first moments they lay eyes on their newborn. These feelings emerge as an intense and unique form of love. But there are also mothers who describe that their maternal affection developed slowly as they got to know the baby. And for a few mothers these feelings do not come at all. We found that rigid expectations coupled with birth complications, loss of agency and negative self-attribution lead to alienation and hinder the development of a maternal embodied emotional connection. How should we understand this?

Merleau-Ponty (2010a) observed that mothers often experience a feeling of strangeness or unreality right after birth. This feeling of strangeness can be understood by considering the transition from “an imaginary infant—to whom all possibilities are open—to the condition of being the real infant who can never realize all the imagined possibilities” (p. 80). Alongside this transition, the woman may experience a feeling of disappointment. He stressed the importance of giving the mother “flexible time, long enough to take possession of the child, to identify with the infant, and to love him as her own” (p. 80). Merleau-Ponty (2010b) suggests that the ability to feel affection is not based on natural instinct, but rather on an active choice to invest ourselves in someone or something, such as a newborn child or motherhood itself.

It [feeling of affection] is created by oath, decision, i.e., promised behavior. But we throw ourselves into it, i.e., we end up feeling, and not only by acting, according to the promise. If we did not feel according to the promise, it is because we have not truly decided or promised, a half-decision is a decision to be double. The true decision results in the feeling. (Merleau-Ponty, 2010b, p. 28)

Consistent with this suggestion, Ruddick (1989) believes that maternal care depends on the mother’s choice to “adopt” and commit to the child. She opposes the idea of a natural fusion between mother and child, and rather underscores the mother’s need for individuation. Taking the child into oneself and experiencing maternal love is not felt to be naturally present from birth, but is rather constituted by the mother by making an active decision. Motherhood requires a commitment, actively taking charge of mothering for the child and investing oneself in it.

A dramatic, medicalized birth or physical illness after birth may delay this process of the mother making an active commitment and investing herself in the child. Having experienced becoming a passive bystander who observes rather than participates creates feelings of helplessness, distance and remoteness. We recognize that sociocultural expectations of instant maternal love clash dramatically with these mothers’ experiences and that this clash often creates feelings of failure, remoteness, shame or guilt. Some mothers with definite expectations for birth, motherhood and the child found the adjustment to reality to be a harsh and difficult process, especially when complications arose. Fantasies about the unborn child, birth and motherhood contributed positively to the development of an emotional connection. When the fantasies were in the form of rigid, detailed plans or expectations, however, they became obstacles to the process of welcoming the actual child and the factual reality as a mother (cf. Besser, Vliegen, Luyten, & Blatt, 2008; Pyszczynski & Greenberg, 1987; Røseth et al., 2011). When encountering a traumatic childbirth, a mother may be well equipped to handle this emotionally and find a path of recovery. However, when her capacity to adapt to such though situations is hindered by a personal approach characterized by rigidity and a high level of perfectionism, recovery may be more difficult (Haga, Lynne, Slinning, & Kraft, 2012).

Merleau-Ponty (1962) can deepen our understanding of why adjustment to motherhood after a troublesome start seemed to be more difficult for mothers with detailed plans and expectations. Laying bare the essentially ambiguous, nebulous nature of our lifeworld, he emphasized that there are always dimensions that elude us. Truth and reality, including the reality of motherly affection, are not absolute, but ambiguous and contingent on the co-constitution of meaning among human beings. Pregnancy and motherhood challenge our ability to endure ambiguity because our embodied existence takes extra hold on us (Adams, 2011). In pregnancy, the woman becomes subject to forces beyond her control, even from within her own body, which becomes alienated from her. Nature takes a “strong hold” on her; she finds herself submerged in it. In pregnancy, birth and childrearing, a woman’s embodied existence as a member of the human species restricts her personal existence. She is in many ways “drawn” to earth, and reminded of her finiteness and future death, and during a dramatic birth much more so than during a normal birth (Yalom, 1980). Nevertheless, as Merleau-Ponty (1962) stresses, our embodied existence does not restrict our freedom as if we were only subject to causal forces. Embodied existence rather has “a significance and express[es] my attitude towards the world” (p. 441). Thus, following Merleau-Ponty, a woman has the freedom to choose how she deals with the ambiguity related to pregnancy, childbirth and motherhood. One way of handling this ambiguity is by attempting to gain control by making detailed plans. Yet, any detailed control over a naturally ambiguous world and future is futile (Yalom, 1980). Even if things go according to plan, the meaning of a situation may have changed. The world, other people, and worse, yourself are beyond your full control. So when the illusion of control collapses, as happens during an unexpectedly troublesome birth, we might find ourselves experiencing meaninglessness, a “life arrest”, as Yalom (1980, p. 420) characterized this. The women in the studies we reviewed found themselves thrown into feeling detached from the situation and the baby. The illusion of certainty and control was uprooted dramatically, which led to a lack of meaning and a sense of remoteness to the child. On the other hand, the embodied connections with the child are not merely constricting. If the mother has the possibility to breastfeed and cuddle her child, and her choice to do so is not thwarted, embodiment can show its soothing face, and positive connections between mother and child are likely to emerge.

## Conclusion

This interpretative synthesis shows that a clash between a woman’s expectations concerning her imagined, unborn child, its birth and early phases of life, on the one hand, and the reality, on the other hand, may initiate a deeply felt struggle or sense of inability to love her child. For some of these women, recovery was gained by taking agency, reclaiming a sense of control by interacting and connecting with the baby. Success in breastfeeding became paramount for many women as it was connected to their image of being a “good mother”, and was a way of making amends for the suffering the baby had endured during the birth. However, if breastfeeding is an expression of mothers’ attempts to regain full control, it may increase the risk of failure as the lack of flexibility makes it difficult to endure the ambiguity of the baby’s signals. The growth of motherly love may involve grieving and letting go of an earlier, inadequate image of the birth, child, or motherhood. An acceptance of the reality of what really happened before, during and after birth may open up for new possibilities and a positive resolution.

Health personnel are encouraged to reflect and capitalize on their professional experience of the uncertainties of pregnancy, birth, and early motherhood in order to help women to incorporate realistic expectations for maternal affection during these ambivalent times. The promotion of a realistic sense of agency and ownership during maternity thus deserves greater attention. The compensating effects of bodily contact between mother and child are to be promoted. All the while, however, health personal should be aware that rigid demands concerning breastfeeding or cuddling, from either mother or personnel, can backfire if it is expected to take a perfect shape. Researchers in this field can contribute more extensive qualitative research on women’s experiences of troubled maternal affection. Here, several other important questions should be explored, such as “how do our cultural norms inform our expectations for maternal affection and motherhood in general?”, “do our western cultural expectations increase women’s ambivalence and their feelings of guilt and shame?”, and “how can we best prepare women for motherhood, reducing the gap between expectations and reality, and the sense of detachment from the child?”
